# Modifiable risk factors associated with prediabetes in men and women: a cross-sectional analysis of the cohort study in primary health care on the evolution of patients with prediabetes (PREDAPS-Study)

**DOI:** 10.1186/s12875-014-0216-3

**Published:** 2015-01-22

**Authors:** Alicia Díaz-Redondo, Carolina Giráldez-García, Lourdes Carrillo, Rosario Serrano, Francisco Javier García-Soidán, Sara Artola, Josep Franch, Javier Díez, Patxi Ezkurra, José Manuel Millaruelo, Mateu Seguí, Javier Sangrós, Juan Martínez-Candela, Pedro Muñoz, Albert Goday, Enrique Regidor

**Affiliations:** Department of Preventive Medicine and Public Health, Hospital General Universitario Gregorio Marañón, Madrid, Spain; Department of Preventive Medicine, Public Health (History of Science), Universidad Complutense de Madrid, Madrid, Spain; La Victoria de Acentejo Health Centre, Santa Cruz de Tenerife, Spain; Martin de Vargas Health Centre, Madrid, Spain; Porriño Health Centre, Pontevedra, Spain; Hereza Health Centre, Madrid, Spain; Raval-Sud Primary Care Team, Barcelona, Spain; Tafalla Health Centre, Navarra, Spain; Zumaia Health Centre, Guipúzcua, Spain; Torrero-La Paz Health Centre, Zaragoza, Spain; Es Castell Basic Health Unit, Islas Baleares, Spain; Yecla Health Centre, Murcia, Spain; Family & Community Medicine Teaching Unit, Cantabria, Spain; Endocrinology & Nutrition Department, Del Mar Hospital, Barcelona, Spain; Consortium for Biomedical Research in Epidemiology & Public Health (CIBER en Epidemiología y Salud Pública - CIBERESP), Madrid, Spain

**Keywords:** Prediabetes, Modifiable risk factors, Primary health care, Men, Women, Spain

## Abstract

**Background:**

Prediabetes is a high-risk state for diabetes development, but little is known about the factors associated with this state. The aim of the study was to identify modifiable risk factors associated with the presence of prediabetes in men and women.

**Methods:**

Cohort Study in Primary Health Care on the Evolution of Patients with Prediabetes (PREDAPS-Study) is a prospective study on a cohort of 1184 subjects with prediabetes and another cohort of 838 subjects without glucose metabolism disorders. It is being conducted by 125 general practitioners in Spain. Data for this analysis were collected during the baseline stage in 2012. The modifiable risk factors included were: smoking habit, alcohol consumption, low physical activity, inadequate diet, hypertension, dyslipidemia, and obesity. To assess independent association between each factor and prediabetes, odds ratios (ORs) were estimated using logistic regression models.

**Results:**

Abdominal obesity, low plasma levels of high-density lipoprotein cholesterol (HDL-cholesterol), and hypertension were independently associated with the presence of prediabetes in both men and women. After adjusting for all factors, the respective ORs (95% Confidence Intervals) were 1.98 (1.41-2.79), 1.88 (1.23-2.88) and 1.86 (1.39-2.51) for men, and 1.89 (1.36-2.62), 1.58 (1.12-2.23) and 1.44 (1.07-1.92) for women. Also, general obesity was a risk factor in both sexes but did not reach statistical significance among men, after adjusting for all factors. Risky alcohol consumption was a risk factor for prediabetes in men, OR 1.49 (1.00-2.24).

**Conclusions:**

Obesity, low HDL-cholesterol levels, and hypertension were modifiable risk factors independently related to the presence of prediabetes in both sexes. The magnitudes of the associations were stronger for men than women. Abdominal obesity in both men and women displayed the strongest association with prediabetes. The findings suggest that there are some differences between men and women, which should be taken into account when implementing specific recommendations to prevent or delay the onset of diabetes in adult population.

## Background

Type 2 diabetes mellitus is a major public health problem, in Europe is estimated that 56.3 million −8.5%- of adult population has diabetes and the prevalence is raising globally [1,2]. Additionally, there are some reasons that exacerbate the problem: the impact of diabetes on morbidity and mortality is well known [1,3,4]; the diagnosis is usually delayed around four to seven years after disease onset largely due to absence of symptoms during the early years of type 2 diabetes [5]; and, 10-20% of persons with type 2 diabetes are found to have developed cardiovascular complications by the date of diagnosis, as a result of the high cardiovascular-disease risk entailed by type 2 diabetes per se and the elevated prevalence of cardiovascular risk factors among these patients [6]. Indeed, this is why the promotion of healthy lifestyles and an early diagnosis constitutes a key strategic line of approach to this health problem.

An appreciable percentage of the population has been observed in the so-called "prediabetic" states. In Spain, the prevalence estimated is above 12% [7]. A subject with prediabetes is defined as any person having blood glucose concentrations that are higher than normal but not of a magnitude which would correspond to a diagnosis of type 2 diabetes [8]. Individuals with prediabetes have an increase high risk of developing type 2 diabetes and associated complications, and most type 2 diabetes patients have likely been in a state of hyperglycemia for several years prior to diagnosis [9]. Prediabetes is also associated with a higher risk of major cardiovascular diseases appearing [10]. On the other hand, there evidence that changes in lifestyle can reduce or delay the appearance of diabetes in prediabetic individuals [11]. Hence, it is important to identify individuals with a higher probability of presenting some glucose metabolism disorder and know the related risk factors.

Some authors assign the same risk factors that have been identified in studies based on type 2 diabetes patients to subjects with prediabetic states [12,13]. Also, it has been suggested that some lifestyle-related risk factors and other component factors of the metabolic syndrome, implicated in the progression of the prediabetic stage to type 2 diabetes, might also contribute to the appearance of prediabetes [13,14]. However, few studies about this issue have been based on prediabetic individuals, and most of these have focused only on metabolic factors [15,16]. Moreover, in the studies that have been undertaken, the authors have tended to analyze the independent effect of these factors, adjusting for sex, thereby rendering it impossible to ascertain whether the factors analyzed display a similar relationship with prediabetes in both sexes. This is why this study aims to identify the modifiable risk factors associated with prediabetes in both men and women.

## Methods

### Study design and subjects

The participants were the 2022 subjects included in the Cohort Study in Primary Health Care on the Evolution of Patients with Prediabetes (Evolución de pacientes con prediabetes en Atención Primaria de Salud - PREDAPS). The background and methodology of this study have been previously published [17]; basically, it consisted of a follow-up study of a cohort of 1184 subjects with prediabetes and another cohort of 838 subjects without glucose metabolism disorders. Baseline data were collected in 2012, in the context of routine clinical practice, by 125 general practitioners distributed across Spain.

Patients who consecutively sought medical attention for any reason and met the inclusion criteria were invited by the researchers to participate in the study. A prediabetic subject was defined as anyone who fulfilled the following criteria: fasting plasma glucose (FPG) levels between 100 and 125 mg/dl, and/or a glycated hemoglobin HbA1c range from 5.7% (39 mmol/mol) to 6.4% (46 mmol/mol) in the preceding 6 months [8]. Patients who had no results for the previous 6 months underwent analysis to determine these values. The selection of a prediabetic patient was matched by the inclusion of another patient of the same sex and similar age (+/− 5 years), in order to form the cohort of subjects without glucose metabolism disorders. The exclusion criteria for both cohorts of subjects were age under 30 or over 74 years, and presence of any of the following processes: diabetes, terminal disease, pregnancy, major surgery or hospital admission in the preceding three months, or any hematologic diseases that might interfere with the HbA1c value.

The study was classified by the Spanish Agency of Medicines and Medical Devices (Agencia Española de Medicamentos y Productos Sanitarios) as a Non-Interventional (Observational) Post-Authorization Study, and the protocol was approved by the Parc de Salut Mar Clinical Research Ethics Committee in Barcelona. All the patients included in the study signed the informed consent form required for their participation.

### Measures

Data on modifiable lifestyle-related risk factors were collected. With respect to smoking habit, subjects were classified into individuals who had smoked at some time in their lives, individuals who currently smoke, and those who had never smoked (ever smoker, current smoker and never smoker). Based on alcohol consumption patterns [18], subjects were classified into risky drinker, low-risk drinker and abstinent categories. Men were considered as risky drinker if they have more than 4 drinks on any single day and/or they have more than 14 drinks per week. Women were considered as risky drinker if they have more than 3 drinks on any single day and/or they have more than 7 drinks per week. Subjects who drink but do not exceed both above limits -any single day and per week- were classified as low-risk drinker. Subjects who do not drink any alcohol were classified as abstinent. Data were obtained on the frequency –number of times in the last two weeks- and amount –mean time in minutes for each session- of different types of physical activity, and, on the basis of the data collected, the minutes per week of physical activity performed by each participant were estimated. Subjects were classified into two categories according to if they had followed or not the World Health Organization (WHO) physical activity recommendations -accumulate at least 150 minutes per week of moderate aerobic activity or 75 minutes per week of vigorous aerobic activity, or an equivalent combination of moderate and vigorous activity- [19].

Participants were asked to describe their usual breakfast, with a wide variety of foods being included as possible replies. Subjects were allocated to two groups, those who had no or an incomplete breakfast (e.g., only coffee, milk, chocolate, cocoa or yogurt), and the rest. Based on the data collected on the frequency of consumption of fruit and vegetables, subjects were classified into the following two categories: those who ate these foods daily, and those who ate them less often. In the case of consumption of fish and legumes, subjects were classified into those who ate these foods three or more times per week, and those who ate them less frequently [20].

Data on blood pressure, height, weight, and waist circumference were obtained by physical examination. Patients were deemed to be hypertensive if their mean systolic pressure -three readings- was ≥140 mmHg, or their mean diastolic pressure was ≥90 mmHg, or if they were undergoing treatment with some type of antihypertensive medication, or if they had personal history of hypertension. General obesity was defined as a Body Mass Index (BMI) ≥30 Kg/m^2^, and abdominal obesity as a waist circumference ≥102 cm in men and ≥88 cm in women [21].

Finally, plasma lipid concentrations were determined, subjects were deemed to have high cholesterol if their levels were ≥250 mg/dl, and high triglycerides if their levels were ≥200 mg/dl; and they were deemed to have low high-density lipoprotein cholesterol (HDL-cholesterol) if their plasma levels were <40 mg/dl in men and <50 mg/dl in women. Other variables, included in this study due to their potential confounding effect with respect to the association studied, were: age, marital status, educational level, region of residence, and family history of diabetes.

### Statistical analyses

We estimated the percentage of patients who presented each of these risk factors in the cohort of subjects with prediabetes and in the cohort without glucose metabolism disorders, with a breakdown by sex. We assessed the statistical significance of the difference in percentages between the two groups using the chi-squared test. A p-value <0.05 were considered statistically significant. In order to evaluate the independent association, in men and in women, between risk factors and presence of prediabetes, logistic regression models were performed. First, we estimated the association between each risk factor and prediabetes using models adjusted for age, marital status, educational level, region of residence, and family history of diabetes. Second, all risk factors were introduced in the models. The estimates were expressed in odds ratios (ORs) and 95% confidence intervals (95% CIs). These analyses were performed using the SPSS statistical package version 19.

## Results

Characteristics of subjects with prediabetes and those without glucose metabolism disorders are shown in Table 1, with a breakdown by sex. Whereas age, percentage of low educational level, and presence of family history of diabetes were higher among subjects with prediabetes, in both men and women, no statistically significant differences were found in terms of marital status or region of residence.

**Table 1:** **Percent distribution of sociodemographic characteristics and family history of diabetes mellitus in men and women**

**Characteristics**	**Men**			**Women**		
	**With prediabetes**	**Without glucose metabolism disorders**	***p*** **-value**	**With prediabetes**	**Without glucose metabolism disorders**	***p*** **-value**
	**N = 595**	**N = 388**		**N = 589**	**N = 450**	
**Age**						
30-49 years	16.5	22.4	0.013	15.1	25.3	<0.001
50-64 years	50.4	51.8		49.1	46.4	
65-74 years	33.1	25.8		35.8	28.2	
**Educational level** ^*^						
Low	59.0	52.3	0.042	70.1	59.6	<0.001
High	41.0	47.7		29.9	40.4	
**Marital status**						
Without partner	15.8	18.3	0.337	27.5	27.1	0.944
Married or with partner	84.2	81.7		72.5	72.9	
**Region of residence** ^**†**^						
North	41.5	39.4	0.131	36.2	36.0	0.955
Centre	30.4	36.3		33.6	34.4	
South	28.1	24.2		30.2	29.6	
**Family history of diabetes** ^**‡**^						
Yes	43.4	33.8	0.003	50.6	34.2	<0.001
No	56.6	66.2		49.4	65.8	

Data are showed in percentages.

^*^Low educational level: lower than secondary (equivalent to less than 12 years of education); high educational level: secondary or higher (equivalent to 12 years or more of education).

^†^North: Galicia, Asturias, Cantabria, Basque Country, Navarre, La Rioja, Aragon, Catalonia; Centre: Castile- León, Madrid, Extremadura, Castile-La Mancha; South: Andalusia, Murcia, Valencia, Balearic Isles, Canary Islands.

^**‡**^Family history of diabetes in father, mother, siblings or children.

The frequency of risk factors in both cohorts of subjects is shown in Table 2. No significant differences in physical activity, diet, and hypercholesterolemia were found. Men and women with prediabetes registered a higher frequency of hypertension, low HDL-Cholesterol level, general obesity, and abdominal obesity than men and women without glucose metabolism disorders. Among men, the percentage of current smokers was lower and the percentage of risky drinkers was higher in the prediabetes group than in the group without glucose metabolism disorders. Women with prediabetes displayed a higher prevalence of hypertriglyceridemia than women without glucose metabolism disorders.

**Table 2:** **Percent distribution of modifiable risk factors present in men and women**

**Risk factors**	**Men**			**Women**		
	**With prediabetes**	**Without glucose metabolism disorders**	***p*** **-value**	**With prediabetes**	**Without glucose metabolism disorders**	***p*** **-value**
	**N = 595**	**N = 388**		**N = 589**	**N = 450**	
**Smoking habit**						
Current smoker	18.7	24.7	0.003	14.6	20.2	0.580
**Alcohol consumption**						
Risky drinker	34.1	31.2	0.015	12.7	12.9	0.469
**Physical activity**						
WHO recommendations not followed	38.4	37.4	0.788	51.2	49.6	0.617
**Diet**						
Incomplete or absent breakfast	16.3	15.7	0.859	10.9	8.0	0.137
Non-daily fruit consumption	26.6	25.8	0.824	17.7	20.0	0.377
Non-daily vegetable consumption	49.6	52.1	0.473	36.7	37.1	0.897
Fish consumed <3 times per week	57.6	61.1	0.289	52.8	54.9	0.530
Legumes consumed <3 times per week	64.4	68.0	0.243	69.1	70.2	0.734
**Blood pressure**						
Hypertension^*^	72.3	51.3	<0.001	61.6	44.2	<0.001
**Lipids**						
Hypercholesterolemia^†^	11.3	12.9	0.481	16.6	14.7	0.440
Hypertriglyceridemia^‡^	14.6	10.4	0.064	10.9	6.2	0.011
Low HDL-Cholesterol^§^	20.4	12.2	0.001	28.3	18.9	0.001
**Obesity**						
General obesity^||^	42.4	23.2	<0.001	43.6	25.1	<0.001
Abdominal obesity^¶^	59.4	33.0	<0.001	75.2	52.2	<0.001

Data are showed in percentages.

^*^Hypertension: mean systolic pressure ≥140 mmHg, or mean diastolic pressure ≥90 mmHg, or being under treatment with antihypertensive drugs, or a personal history of arterial hypertension.

^†^Hypercholesterolemia: plasma total cholesterol ≥250 mg/dl.

^‡^Hypertriglyceridemia: plasma triglycerides ≥200 mg/dl.

^§^Low HDL-Cholesterol: plasma High-Density Lipoprotein cholesterol <40 mg/dl in men, and <50 mg/dl in women.

^||^General obesity: Body Mass Index ≥30 kg/m^2^.

^¶^Abdominal obesity: waist circumference ≥102 cm in men, and ≥88 cm in women.

The ORs that reflect the association between each of the risk factors and presence of prediabetes in men and in women are shown in Tables 3 and 4, respectively. In model 1, the ORs -adjusted for age, educational level, marital status, region of residence, and family history of diabetes- showed an elevated magnitude for hypertension, low HDL-Cholesterol, general obesity, and abdominal obesity in men and women alike. In men, risky alcohol consumption and hypertrigliceridemia also yielded elevated and significant ORs. In women, incomplete or absent breakfast and hypertrigliceridemia were elevated but no significant OR.

**Table 3:** **Association between modifiable risk factors and presence of prediabetes in men**

**Risk factors**	**Model 1**		**Model 2**	
	**OR**	**(95% CI)**	**OR**	**(95% CI)**
**Smoking habit**				
Current smoker vs. never smoker	0.94	(0.65–1.37)	0.77	(0.51–1.16)
**Consumption of alcohol**				
Risky drinker vs. abstinent	**1.58**	**(1.09–2.28)**	**1.49**	**(1.00–2.24)**
**Physical activity**				
WHO recommendations not followed vs. followed	1.13	(0.86–1.48)	1.10	(0.82–1.49)
**Breakfast**				
Incomplete or absent vs. complete	1.12	(0.78–1.60)	1.10	(0.75–1.63)
**Consumption of fruit**				
Non-daily vs. daily consumption	1.16	(0.86–1.57)	1.26	(0.89–1.77)
**Consumption of vegetables**				
Non-daily vs. daily consumption	0.96	(0.74–1.25)	0.88	(0.65–1.19)
**Consumption of fish**				
Consumption of <3 vs. >3 times per week	0.88	(0.67–1.16)	0.91	(0.68–1.23)
**Consumption of legumes**				
Consumption of <3 vs. >3 times per week	0.87	(0.66–1.14)	0.84	(0.63–1.13)
**Blood pressure**				
Hypertension^*^ vs. no hypertension	**2.33**	**(1.76–3.08)**	**1.86**	**(1.39–2.51)**
**Lipids**				
Hypercholesterolemia^†^ vs. no hypercholesterolemia	0.85	(0.57–1.27)	0.99	(0.64–1.53)
Hypertriglyceridemia^‡^ vs. no hypertriglyceridemia	**1.61**	**(1.07–2.42)**	1.19	(0.75–1.90)
Low HDL-Cholesterol^§^ vs. high HDL-Cholesterol	**1.94**	**(1.34–2.82)**	**1.88**	**(1.23–2.88)**
**Obesity**				
General obesity^||^ vs. no general obesity	**2.51**	**(1.88–3.36)**	1.43	(0.99–2.06)
Abdominal obesity^¶^ vs. no abdominal obesity	**2.87**	**(2.19–3.77)**	**1.98**	**(1.41–2.79)**

Data are showed in odds ratios (OR) and 95% confidence intervals (95% CI). Model 1 is adjusted for age, educational level, marital status, region of residence, and family history of diabetes. Model 2 is adjusted for all the variables in the table plus age, educational level, marital status, region of residence, and family history of diabetes. Data in bold indicate statistically significant associations.

^*^Hypertension: mean systolic pressure ≥140 mmHg, or mean diastolic pressure ≥90 mmHg, or being under treatment with antihypertensive drugs, or a personal history of arterial hypertension.

^**†**^Hypercholesterolemia: plasma total cholesterol ≥250 mg/dl.

^‡^Hypertriglyceridemia: plasma triglycerides ≥200 mg/dl.

^§^Low HDL-Cholesterol: plasma High-Density Lipoprotein cholesterol <40 mg/dl.

^||^General obesity: Body Mass Index ≥30 kg/m^2^.

^¶^Abdominal obesity: waist circumference ≥102 cm.

**Table 4:** **Association between modifiable risk factors and presence of prediabetes in women**

**Risk factors**	**Model 1**		**Model 2**	
	**OR**	**(95% CI)**	**OR**	**(95% CI)**
**Smoking habit**				
Current smoker vs. never smoker	0.82	(0.57–1.17)	0.75	(0.50–1.11)
**Consumption of alcohol**				
Risky drinker vs. abstinent	1.00	(0.66–1.50)	1.13	(0.72–1.76)
**Physical activity**				
WHO recommendations not followed vs. followed	1.15	(0.89–1.48)	1.04	(0.79–1.37)
**Breakfast**				
Incomplete or absent vs. complete	1.48	(0.95–2.30)	1.43	(0.89–2.30)
**Consumption of fruit**				
Non-daily vs. daily consumption	1.06	(0.76–1.48)	0.94	(0.65–1.37)
**Consumption of vegetables**				
Non-daily vs. daily consumption	0.98	(0.75–1.27)	0.93	(0.70–1.24)
**Consumption of fish**				
Consumption of <3 vs. >3 times per week	0.95	(0.73–1.23)	0.94	(0.71–1.26)
**Consumption of legumes**				
Consumption of <3 vs. >3 times per week	0.94	(0.71–1.23)	0.88	(0.66–1.19)
**Blood pressure**				
Hypertension^*^ vs. no hypertension	**1.79**	**(1.37–2.35)**	**1.44**	**(1.07–1.92)**
**Lipids**				
Hypercholesterolemia^†^ vs. no hypercholesterolemia	1.04	(0.74–1.48)	1.13	(0.78–1.65)
Hypertriglyceridemia^‡^ vs. no hypertriglyceridemia	1.58	(0.98–2.54)	1.08	(0.64–1.80)
Low HDL-Cholesterol^§^ vs. high HDL-Cholesterol	**1.82**	**(1.33–2.49)**	**1.58**	**(1.12–2.23)**
**Obesity**				
General obesity^||^ vs. no general obesity	**2.26**	**(1.71–2.99)**	**1.42**	**(1.01–1.98)**
Abdominal obesity^¶^ vs. no abdominal obesity	**2.55**	**(1.94–3.35)**	**1.89**	**(1.36–2.62)**

Data are showed in odds ratios (OR) and 95% confidence intervals (95% CI). Model 1 is adjusted for age, educational level, marital status, region of residence, and family history of diabetes. Model 2 is adjusted for all the variables in the table plus age, educational level, marital status, region of residence, and family history of diabetes. Data in bold indicate statistically significant associations.

^*^Hypertension: mean systolic pressure ≥140 mmHg, or mean diastolic pressure ≥90 mmHg, or being under treatment with antihypertensive drugs, or a personal history of arterial hypertension.

^†^Hypercholesterolemia: plasma total cholesterol ≥250 mg/dl.

^‡^Hypertriglyceridemia: plasma triglycerides ≥200 mg/dl.

^§^Low HDL-Cholesterol: plasma High-Density Lipoprotein cholesterol <50 mg/dl.

^||^General obesity: Body Mass Index ≥30 kg/m^2^.

^¶^Abdominal obesity: waist circumference ≥ 88 cm.

After adjustment for all risk factors (model 2), hypertension, low HDL-Cholesterol plasma levels and abdominal obesity showed an independent association with prediabetes in men and women. Furthermore, alcohol consumption showed an independent association in men, and general obesity showed an independent association in women.

## Discussion

This study showed that known cardiovascular risk factors, specifically abdominal obesity, hypertension and low HDL-Cholesterol levels, are independently associated with the presence of prediabetes in women and men alike. Additionally, general obesity in women and alcohol consumption in men are risk factors for prediabetes. General obesity was also associated with prediabetes in men, although the statistical significance disappeared after adjustment for all factors. The modifiable risk factors that showed the strongest association with prediabetes was abdominal obesity. The magnitudes of the associations were stronger for men than women. Smoking, physical activity, and diet habits not showed significant relationship with prediabetes.

As other studies, obesity in our study subjects –both general and, more specifically, abdominal– was associated with the presence of prediabetes [7,22]. Moreover, when a wide number of risk factors had been examined simultaneously, as we have done in our study, waist circumference had showed the strongest direct effect on prediabetes [22]. Although the mechanisms are not clear, excess adipose tissue has been reported to release fatty acids, something that induces resistance to insulin in the muscle and, in turn, leads to elevation of plasma glucose levels [13]. Also, measures of abdominal obesity have been found as the strongest predictors of diabetes [23].

The association between hypertension/dyslipidemia and prediabetes has been reported previously [7,14,24]. In our study, hypertension displayed an important association with prediabetes, particularly among men. Hypertriglyceridemia and low HDL-Cholesterol levels were also seen to be associated with prediabetes, though only the association with low HDL-Cholesterol levels proved to be significant in both sexes. Moreover, when the rest variables had been adjusted for, the magnitude of the association between these factors and prediabetes fell substantially in both sexes, though hypertension and low HDL-Cholesterol levels remain as independent risk factors for prediabetes. This weakening, in the association between dyslipidemia and hypertension with prediabetes, is basically due to the inclusion of obesity in the model. This is supported by the strong association existing between obesity and blood pressure, although the mechanisms are not yet fully known [25]. Also, Bardenheier et al. found in their model that the direct effect of hypertension on prediabetes was confounded by waist circumference [22].

A number of studies have reported that alcohol consumption in men, and excessive consumption in particular, is associated with the appearance of prediabetes and type 2 diabetes, whereas alcohol consumption in women displays a protective effect for the appearance of these health problems [26,27]. In our study, alcohol consumption was found as a modifiable risk factor for prediabetes in men. Likewise, a lower frequency of risky drinkers was observed among women with prediabetes than among those without glucose disorders, but the differences were not significant.

It has noted that frequency and quality of breakfast is related with regulation of appetite and control of plasma glucose levels [28]. Also, positive associations between skipping breakfast and fasting glucose and fasting insulin have been observed [29]. In our study, although the magnitude of this association was high in women, no statistically significant relationship was found between absent or incomplete breakfast and prediabetes. On the other hand, no relationship was found between consumption of other foods and the presence of prediabetes. This is consistent with the observed by others in relation to fruit and vegetables consumption [14], but it is contrary to the protective effect of foods such as fish, vegetables and legumes on glucose disorders that has been reported [30].

Although the physical exercise is known as a protective factor for the development of type 2 diabetes [31], the failure to follow the WHO physical-activity recommendations was not shown to be associated with the presence of prediabetes in our study. Other studies have obtained similar findings [14]. In relation to smoking, it is a known risk factor of diabetes [32]; however, there is inconsistent evidence about prediabetes. Some findings are agreed with ours [14], but in others smoking was reported as a risk factor [33]. The criteria of prediabetes used in above studies differ from those we used in our study, and therefore, the groups formed in each study are not fully comparable to each other.

### Strengths and limitations

The main strength of the present study is the focus on the prediabetes state. Also, we have included a large number of participants from across Spain from primary care setting. However, when it comes to interpreting the findings, some limitations must be borne in mind. First, this study should be consider as a cross-sectional in design, since it was based on data collected during the baseline stage of a follow-up study. Hence, one cannot exclude the possibility that subjects with prediabetes might have modified their lifestyles and acquired healthier habits. Second, this study included subjects with prediabetes based on HbA1c and FPG criteria, the exclusion of oral glucose overload as criteria for prediabetes was due to it is an expensive and impractical procedure at a primary health-care level. The two criteria used (FPG and HbA1c) have been widely accepted by the American Diabetes Association [8]. Third, the number of patients included in the prediabetes cohort is greater than the number of patients included in the cohort of subjects without glucose metabolism disorders. Many physicians included patients with normal FPG levels in the second cohort without waiting for the result of the HbA1c analytical tests, when the HbA1c results arrived, some patients were found to be prediabetic and were changed to the prediabetes cohort. Fourth, in our study subjects were health service users, this mean that it would be expected that subjects without glucose metabolism disorders display a higher frequency of risk factors than would the general population; thus, the possibility of identifying risk factors associated with prediabetes would be correspondingly lower than in population-based studies.

## Conclusions

This study provides useful information when considering early detection of individuals with high risk of development diabetes and cardiovascular diseases. The detection of prediabetes in an individual should be accompanied by interventions addressing modifiable risk factors. Obesity, hypertension and low HDL-cholesterol levels have been the modifiable risk factors independently related to the presence of prediabetes identified in this study; also, alcohol consumption in men. The magnitudes of the associations were stronger for men than women. Abdominal obesity in both men and women displayed the strongest association with prediabetes.

The available evidence is still scarce, further studies are needed to check these findings and to confirm possible explanations. The existence of differences between men and women underscore the need to analyze data separately by sex, in order to implement specific recommendations to primary prevention of diabetes and cardiovascular diseases in adult population. Finally, these results might influence the primary care practice where, for example, in men, the alcohol consumption should be considered as an additional risk factor of prediabetes compared to women.

## References

[1] International Diabetes Federation (2013). IDF Diabetes Atlas.

[2] Danaei G, Finucane MM, Lu Y, Singh GM, Cowan MJ, Paciorek CJ, Lin JK, Farzadfar F, Khang Y-H, Stevens GA, Rao M, Ali MK, Riley LM, Robinson CA, Ezzati M (2011). National, regional, and global trends in fasting plasma glucose and diabetes prevalence since 1980: systematic analysis of health examination surveys and epidemiological studies with 370 country-years and 2.7 million participants. Lancet.

[3] Jansson SPO, Andersson DKG, Svärdsudd K (2010). Mortality trends in subjects with and without diabetes during 33 years of follow-up. Diabetes Care.

[4] Wermeling PR, Gorter KJ, van Stel HF, Rutten GE. Both cardiovascular and non-cardiovascular comorbidity are related to health status in well-controlled type 2 diabetes patients: a cross-sectional analysis. Cardiovasc Diabetol. 2012;11:121. 10.1186/1475-2840-11-121 PMC3508839 23039172

[5] Harris MI, Klein R, Welborn TA, Knuiman MW (1992). Onset of NIDDM occurs at least 4–7 yr before clinical diagnosis. Diabetes Care.

[6] Medrano MJ, Pastor-Barriuso R, Boix R, del Barrio JL, Damián J, Alvarez R, Marín A (2007). Coronary disease risk attributable to cardiovascular risk factors in the Spanish population. Rev Esp Cardiol.

[7] Soriguer F, Goday A, Bosch-Comas A, Bordiú E, Calle-Pascual A, Carmena R, Casamitjana R, Castaño L, Castell C, Catalá M, Delgado E, Franch J, Gaztambide S, Girbés J, Gomis R, Gutiérrez G, López-Alba A, Martínez-Larrad MT, Menéndez E, Mora-Peces I, Ortega E, Pascual-Manich G, Rojo-Martínez G, Serrano-Rios M, Valdés S, Vázquez JA, Vendrell J, Casamitjana R, Castaño L, Castell C (2012). Prevalence of diabetes mellitus and impaired glucose regulation in Spain: The Di@bet.es Study. Diabetologia.

[8] American Diabetes Association (2011). Standards of medical care in diabetes-2011. Diabetes Care.

[9] Aroda VR, Ratner R (2008). Approach to the patient with prediabetes. J Clin Endocrinol Metab.

[10] DeFronzo RA, Abdul-Ghani M (2011). Assessment and treatment of cardiovascular risk in prediabetes: impaired glucose tolerance and impaired fasting glucose. Am J Cardiol.

[11] Tuomilehto J, Lindström J, Eriksson JG, Valle TT, Hämäläinen H, Ilanne-Parikka P, Keinänen-Kiukaanniemi S, Laakso M, Louheranta A, Rastas M, Salminen V, Uusitupa M (2001). Prevention of type 2 diabetes mellitus by changes in lifestyle among subjects with impaired glucose tolerance. N Engl J Med.

[12] Nathan DM, Davidson MB, DeFronzo RA, Heine RJ, Henry RR, Pratley R, Zinman B (2007). Impaired fasting glucose and impaired glucose tolerance: implications for care. Diabetes Care.

[13] Grundy SM (2012). Pre-diabetes, metabolic syndrome, and cardiovascular risk. J Am Coll Cardiol.

[14] Khambalia A, Phongsavan P, Smith BJ, Keke K, Dan L, Fitzhardinge A, et al. Prevalence and risk factors of diabetes and impaired fasting glucose in Nauru. BMC Public Health. 2011;11:719. 10.1186/1471-2458-11-719 PMC3187757 21943388

[15] Nóvoa FJ, Boronat M, Saavedra P, Díaz-Cremades JM, Varillas VF, La Roche F, Alberiche MP, Carrillo A (2005). Differences in cardiovascular risk factors, insulin resistance, and insulin secretion in individuals with normal glucose tolerance and in subjects with impaired glucose regulation: The Telde Study. Diabetes Care.

[16] Weyer C, Bogardus C, Pratley RE (1999). Metabolic characteristics of individuals with impaired fasting glucose and/or impaired glucose tolerance. Diabetes.

[17] Serrano R, García-Soidán FJ, Díaz-Redondo A, Artola S, Franch J, Díez J, Carrillo L, Ezkurra P, Millaruelo JM, Seguí M, Sangrós FJ, Martínez-Candela J, Muñoz P, Goday A, Regidor E (2013). Cohort study in primary health care on the evolution of patients with prediabetes (PREDAPS): basis and methodology. Rev Esp Salud Pública.

[18] National Institute on Alcohol Abuse and Alcoholism (2010). Rethinking Drinking: Alcohol and Your Health.

[19] World Health Organization (2010). Global recommendations on physical activity for health.

[20] Martínez-González MA, García-Arellano A, Toledo E, Salas-Salvadó J, Buil-Cosiales P, Corella D, et al. A 14-item Mediterranean diet assessment tool and obesity indexes among high-risk subjects: The PREDIMED trial. PloS One. 2012;7:e43134. 10.1371/journal.pone.0043134 PMC3419206 22905215

[21] Salas-Salvadó J, Rubio MA, Barbany M, Moreno B, Grupo Colaborativo de la SEEDO (2007). SEEDO 2007 Consensus for the evaluation of overweight and obesity and the establishment of therapeutic intervention criteria. Med Clin (Barc).

[22] Bardenheier BH, Bullard KM, Caspersen CJ, Cheng YJ, Gregg EW, Geiss LS (2013). A novel use of structural equation models to examine factors associated with prediabetes among adults aged 50 years and older: National Health and Nutrition Examination Survey 2001–2006. Diabetes Care.

[23] Zhao X, Zhu X, Zhang H, Zhao W, Li J, Shu Y, et al. Prevalence of diabetes and predictions of its risks using anthropometric measures in southwest rural areas of China. BMC Public Health. 2012;12:821. 10.1186/1471-2458-12-821 PMC3549931 22998969

[24] Heianza Y, Hara S, Arase Y, Saito K, Fujiwara K, Tsuji H, Kodama S, Hsieh SD, Mori Y, Shimano H, Yamada N, Kosaka K, Sone H (2011). HbA1c 5.7-6.4% and impaired fasting plasma glucose for diagnosis of prediabetes and risk of progression to diabetes in Japan (TOPICS 3): a longitudinal cohort study. Lancet.

[25] Kang YS (2013). Obesity associated hypertension: new insights into mechanism. Electrolyte Blood Press.

[26] Cullmann M, Hilding A, Östenson C-G (2012). Alcohol consumption and risk of pre-diabetes and type 2 diabetes development in a Swedish population. Diabet Med.

[27] Liu C, Yu Z, Li H, Wang J, Sun L, Qi Q, et al. Associations of alcohol consumption with diabetes mellitus and impaired fasting glycemia among middle-aged and elderly Chinese. BMC Public Health. 2010;10:713. 10.1186/1471-2458-10-713 PMC2998495 21092093

[28] Pereira MA, Erickson E, McKee P, Schrankler K, Raatz SK, Lytle LA, Pellegrini AD (2011). Breakfast frequency and quality may affect glycemia and appetite in adults and children. J Nutr.

[29] Smith KJ, Gall SL, McNaughton SA, Blizzard L, Dwyer T, Venn AJ (2010). Skipping breakfast: longitudinal associations with cardiometabolic risk factors in the Childhood Determinants of Adult Health Study. Am J Clin Nutr.

[30] Feskens EJ, Virtanen SM, Räsänen L, Tuomilehto J, Stengård J, Pekkanen J, Nissinen A, Kromhout D (1995). Dietary factors determining diabetes and impaired glucose tolerance. A 20-year follow-up of the Finnish and Dutch cohorts of the Seven Countries Study. Diabetes Care.

[31] Laaksonen DE, Lindström J, Lakka TA, Eriksson JG, Niskanen L, Wikström K, Aunola S, Keinänen-Kiukaanniemi S, Laakso M, Valle TT, Ilanne-Parikka P, Louheranta A, Hämäläinen H, Rastas M, Salminen V, Cepaitis Z, Hakuma M, Kaikkonen H, Härkönen P, Sundvall J, Tuomilehto J, Uusitapa M (2005). Physical activity in the prevention of type 2 diabetes: The Finnish diabetes prevention study. Diabetes.

[32] Willi C, Bodenmann P, Ghali WA, Faris PD, Cornuz J (2007). Active smoking and the risk of type 2 diabetes: a systematic review and meta-analysis. JAMA.

[33] Vlassopoulos A, Lean MEJ, Combet E. Influence of smoking and diet on glycated haemoglobin and “pre-diabetes” categorisation: a cross-sectional analysis. BMC Public Health. 2013;13:1013. 10.1186/1471-2458-13-1013 PMC4029457 24499114

